# *Bacillus velezensis* SQR9 promotes plant growth through colonization and rhizosphere–phyllosphere bacteria interaction

**DOI:** 10.1111/1758-2229.13250

**Published:** 2024-04-04

**Authors:** Zhao Yu, Dongsheng Wang, Bo Zhang, Hancheng Mao, Zhe Wang, Zhiguang Yan, Chengyuan Tao, Xuhui Deng, Qirong Shen, Rong Li

**Affiliations:** ^1^ The Sanya Institute of Nanjing Agricultural University, Jiangsu Provincial Key Lab for Solid Organic Waste Utilization, Key Lab of Organic‐Based Fertilizers of China, Jiangsu Collaborative Innovation Center for Solid Organic Wastes, Educational Ministry Engineering Center of Resource‐saving Fertilizers Nanjing Agricultural University Nanjing Jiangsu People's Republic of China; ^2^ Nanjing Institute of Vegetable Science Nanjing Jiangsu People's Republic of China

## Abstract

The rhizosphere and phyllosphere of plants are home to a diverse range of microorganisms that play pivotal roles in ecosystem services. Consequently, plant growth‐promoting bacteria (PGPB) are extensively utilized as inoculants to enhance plant growth and boost productivity. Despite this, the interactions between the rhizosphere and phyllosphere, which are influenced by PGPB inoculation, have not been thoroughly studied to date. In this study, we inoculated *Bacillus velezensis* SQR9, a PGPB, into the bulk soil, rhizosphere or phyllosphere, and subsequently examined the bacterial communities in the rhizosphere and phyllosphere using amplicon sequencing. Our results revealed that PGPB inoculation increased its abundance in the corresponding compartment, and all treatments demonstrated plant growth promotion effects. Further analysis of the sequencing data indicated that the presence of PGPB exerted a more significant impact on bacterial communities in both the rhizosphere and phyllosphere than in the inoculation compartment. Notably, the PGPB stimulated similar rhizosphere‐beneficial microbes regardless of the inoculation site. We, therefore, conclude that PGPB can promote plant growth both directly and indirectly through the interaction between the rhizosphere and phyllosphere, leading to the enrichment of beneficial microorganisms.

## Graphical abstract

The colonization of PGPB in rhizosphere and phyllosphere both increased plant growth; The colonization of PGPB could shape the bacterial communities of all compartments; Similar beneficial microbes were stimulated by the PGPB even they were not in touch; Plant recruitment induced by PGPB may contributed the enrichment of beneficial microbes.

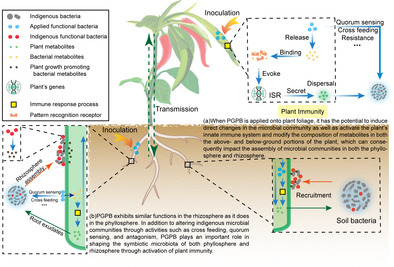

## INTRODUCTION

The plant microbial community, also known as the plant microbiota (including all plant‐related microorganisms), is significant in supporting plant growth, health and productivity (Lemanceau et al., 2017; Trivedi et al., 2020). The interaction between plants and microbial communities is an important driver for maintaining agricultural ecosystems (Vejan et al., 2016). The rhizosphere is a microbial hot spot for numerous plants and is considered one of the most complex ecosystems on Earth (Orozco‐Mosqueda et al., 2018). Most rhizosphere microorganisms can benefit plants through mechanisms involving nutrient solubilization, plant hormone production and pathogen‐antagonistic activity (Vejan et al., 2016). To date, many agricultural practices have been proposed to manipulate rhizosphere microbiome communities to boost plant growth by encouraging beneficial symbionts and plant growth‐promoting bacteria (PGPB) (Balderas‐Ruíz et al., 2020). In comparison, the phyllosphere represents the largest microbial habitat on earth and can also be colonized by plant‐beneficial microorganisms (Ren et al., 2014). The phyllosphere refers to the community of microorganisms that live under symbiotic relationship with plants in the aerial parts like the leaves, and these microorganisms live both on surfaces of plant organs (usually referred to as phylloplane) or inside plant tissues (endosphere) (Carvalho & Castillo, 2018). Although some studies have noted that foliar application of potentially beneficial bacteria can promote plant growth (Esitken et al., 2006), the functional mechanism by which foreign beneficial bacteria inhabit the phyllosphere has been less intensively studied (Shakir et al., 2021; Wang et al., 2019). Rhizosphere–phyllosphere interactions play roles in plant growth and shaping the plant microbial community and are normally overlooked (Aziz et al., 2021). Therefore, exploring how foliar‐applied PGPB promotes plant growth could help us better understand phyllosphere functional microbes.

To explore whether PGPB inoculation of the phyllosphere produces a plant promotional effect similar to that of bulk soil and rhizosphere inoculations, we hypothesized that PGPB could directly colonize the phyllosphere and indirectly stimulate beneficial microorganisms to promote plant growth. Because *Bacillus* is one of the most studied and commonly used PGPB in actual production (Saxena et al., 2020), we selected a well‐studied PGPB strain, *Bacillus velezensis* SQR9 (SQR9), which was originally isolated from crop rhizosphere, as a model PGPB in this study (Huang et al., 2022). To test this hypothesis, we performed a pot experiment. First, *B. velezensis* SQR9 was added to the bulk soil or rhizosphere soil or sprayed on the pepper phyllosphere. We subsequently investigated pepper biomass and the bacterial community of the rhizosphere and phyllosphere through 16S rRNA gene sequencing. Overall, we demonstrated the plant promotional effect and associated mechanisms manipulated by phyllosphere inoculation with PGPB.

## EXPERIMENTAL PROCEDURES

### Description of the pot experiment

Barren soil that had not been fertilized for many years was collected for this study. The main characteristics of the soil were as follows: pH, 7.1; electrical conductivity, 20 μs cm^−1^; ammonium nitrogen, 2.2 mg kg^−1^; and nitrate nitrogen, 14.9 mg kg^−1^. We mixed the barren soil with 2% (DW) commercial organic fertilizer (organic matter content, 450 g kg^−1^; total nitrogen, 17.5 g kg^−1^; total phosphorous, 8.2 g kg^−1^ and total potassium, 14.3 g kg^−1^). The pot experiment contained four treatments: CK (no PGPB inoculation), in which 10 mL clear water was applied to each hole of the seedling tray, 50 mL clear water was sprayed on the leaves and 500 mL of clear water was poured on the soil during potting; R9 (rhizosphere inoculated with SQR9), in which 10 mL of SQR9 bacterial solution (with a concentration of 10^8^ cfu mL^−1^) was applied to each hole of the seedling tray, 50 mL clear water was sprayed on the leaves and 500 mL of clear water was poured on the soil during potting; L9 (leaf inoculated with SQR9), in which 10 mL clear water was applied to each hole of the seedling tray, 50 mL of SQR9 bacterial solution (with a concentration of 10^8^ cfu mL^−1^) was sprayed on the leaves, and 500 mL of clear water was poured on the soil during potting; and B9 (bulk soil inoculated with SQR9), in which 10 mL clear water was applied to each hole of the seedling tray, 50 mL clear water was sprayed on the leaves and 500 mL of SQR9 bacterial solution (with a concentration of 10^8^ cfu mL^−1^) was poured on the soil during potting (Figure S1).

The peppers (*Capsicum annuum* L.) used in this experiment were the commercial cultivar ‘Su Jiao Wu Hao Bo Shi Wang’, which is an early ripening pepper cultivar with a long lantern shape and a light green, glossy surface to the fruit. On the fifth day after germination, the seedlings were transferred to a 54‐hole nursery tray containing a nursery substrate. On Day 12, the seedling substrate was treated with *B. velezensis* SQR9. On Day 21, the pepper seedlings were transplanted into pots. Water was applied every 3 days or when the soil was dry after the transplantation of pepper seedlings and until the end of the pot experiment. On Day 35, the bulk soil was irrigated with SQR9, and the leaves were sprayed with SQR9. During spraying, a shield was used to ensure that the roots and soil were not contacted by the liquid. The plant height and stem diameter of pepper plants were measured on Day 56. Each treatment contained 10 replicates that were randomly placed and periodically replaced during pot planting to eliminate environmental impacts.

### Sample collection

Soil and leaf sampling were performed for subsequent analysis on Day 56. For all treatments, six healthy plants with uniform growth were randomly selected to collect rhizosphere soil and leaf samples. For the rhizosphere soil, entire healthy plants were collected and shaken vigorously to remove excess soil, and the soil still adhering to the roots was considered to be rhizosphere soil (Fu et al., 2017). Next, the collected crop roots were cut into 1 cm lengths with sterile scissors, mixed well, placed into a conical flask containing glass beads (3 mm in diameter) and 250 mL of sterile physiological saline (0.90%, NaCl), and shaken on a shaker at 170 r min^−1^ at room temperature for 30 min. The roots were then ultrasonically treated for 5 min, the root system was removed with sterile tweezers, the soil suspension was centrifuged at 10,000 for 10 min, the supernatant was discarded and the precipitate was the rhizosphere soil, which was subsequently stored at −80°C for DNA extraction. For the leaf samples, nine leaves from each plant were randomly selected and mixed as a representative sample. After sampling, the surface of each leaf was washed with water and dried with absorbent paper. The leaves were then stored in liquid nitrogen and transported to the laboratory within 4 h for subsequent processing. In the laboratory, the leaves were immersed in liquid nitrogen, quickly ground, placed in a 2 mL sterile centrifuge tube and stored at −80°C for DNA extraction.

### DNA extraction and MiSeq high‐throughput sequencing

The sample DNA was extracted using the PowerSoil Soil DNA Isolation Kit (MoBio Laboratories Inc., USA) following the manufacturer's protocol. MiSeq high‐throughput sequencing of rhizobacteria and phyllosphere bacteria followed the method published by Caporaso et al. (2011). The V5‐V6 region of the bacterial 16S rRNA gene was amplified from leaf genomic DNA with primers 799F (5′‐AAC MGG ATT AGA TAC CCK G‐3′) and 1115R (5′‐AGG GTT GCG CTC GTT G‐3′). All PCR amplification, library preparation and onboard sequencing were carried out at Shanghai Personalbio Technology Co., Ltd.

### Data quality control

First, the high‐throughput sequencing data were quality‐controlled and annotated according to the quality control process of Liu et al. (2016). QIIME software was used to assign the sequence of each sample to the corresponding barcode information, remove the adapter and primer sequences for the original sequence after disembarkation and remove low‐quality sequences, after which the forward and reverse sequences were spliced (Caporaso et al., 2010). The spliced sequences were analysed for high‐throughput data according to the standard analysis process of UPARSE, and an operational taxonomic unit (OTU) table was generated to select representative sequences for each OTU (Edgar, 2013). For the pot experiments, this step generated a 16S rRNA OTU table of 48 samples × 7814 OTUs (3,768,783 reads). The number of high‐quality sequences per sample was 2391–245,555. Finally, classification of the representative sequences for each OTU was performed using the RDP classifier (Wang et al., 2007). To obtain an equivalent sequencing depth for further bacterial community analysis, each sample was rarefied to the smallest sample size (2391 reads) in R using the package GUNIFRAC (function: rarefy).

### Bioinformatics and statistical analysis

All statistical tests performed in this study were considered significant at *p* ≤ 0.05. Alpha diversity indicators such as richness (Sobs) and evenness (Shannon) were determined for each sample. The richness and evenness of rarefied OTUs were calculated using the VEGAN (function: diversity) package in R (v.4.1.2 for Windows). The weighted UniFrac distances between treatments were calculated using the R package GUNIFRAC and presented based on a principal coordinate analysis (PCoA) using the GGPLOT2 package to visualize the differences in microbial community composition. Differences in bacterial community composition among treatments were tested using a permutational multivariate analysis of variance (PERMANOVA), which was performed using the R package “vegan” (function: adonis) with 999 permutations. Variance decomposition (VPA) was calculated using the R package “vegan”; phylum‐ and genus‐level difference analysis of bacteria among different treatments was performed using STAMP (v.2.1.1.0 for Windows). Other statistical analyses such as the LSD test and Tukey's method were conducted using the IBM SPSS 23.0 software program (SPSS Inc., USA).

## RESULTS

**FIGURE 1:**
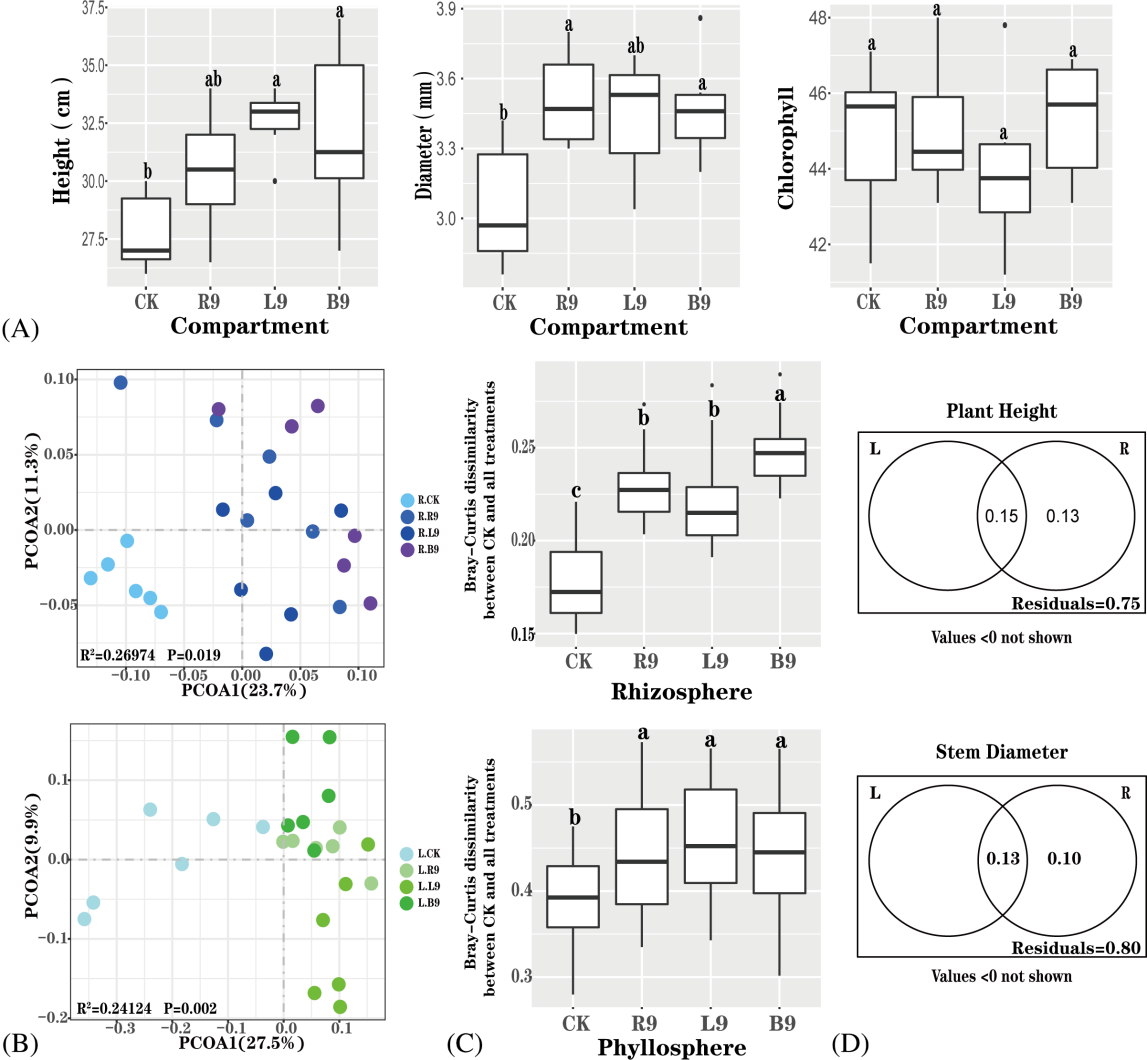
The influence of SQR9 application on plant biomass and the bacterial community. (A) The plant height, stem diameter and chlorophyll content of pepper (mean ± SE, *n* = 6); (B) principal coordinate analysis (PCoA) of the bacterial community based on weighted UniFrac distance, *p*‐values were obtained via PERMANOVA; (C) community dissimilarity based on Bray–Curtis distance, the distances between CK and all treatments are shown; (D) variance partitioning analysis (VPA) explaining the variance in plant height and stem diameter related to rhizosphere and phyllosphere bacterial community composition. CK, treatment without SQR9 application; R9, seedling growing medium was applied with SQR9; L9, leaves sprayed with SQR9; B9, SQR9 added to bulk soil. R, rhizosphere; L, phyllosphere. Different letters indicate significant differences among the treatments as determined by Tukey's test (*p* ≤ 0.05).

The plant height and stem diameter in the treatments with SQR9 were significantly higher than those in the CK, whereas the chlorophyll content did not show a significant difference among treatments. Moreover, among the treatments with SQR9, the application of SQR9 on the foliage showed a larger impact on the plant height and stem diameter than on those of the other treatments, although there were no significant differences among these treatments (Figure 1A). The application of PGPB increased SQR9 abundance in the corresponding compartment compared with that of the CK; however, significant differences were observed only in the phyllosphere application (Figure S2a, S2b and S2c). With regard to bacterial diversity, both the richness and evenness in the rhizosphere and phyllosphere decreased compared with those in CK (Figure S3a, S3b, S3c and S3d). We then performed a PCoA based on the weighted UniFrac distance to visualize the differences in community composition. The results showed that the application of SQR9 significantly changed the bacterial composition in both the phyllosphere and rhizosphere compared with that in the CK (Figure 1B). Similar results were observed in the community dissimilarity analysis between the CK and the other treatments (Figure 1C). Moreover, the rhizobacterial composition explained more of the variance in plant biomass than the phyllosphere bacterial composition (Figure 1D).

**FIGURE 2:**
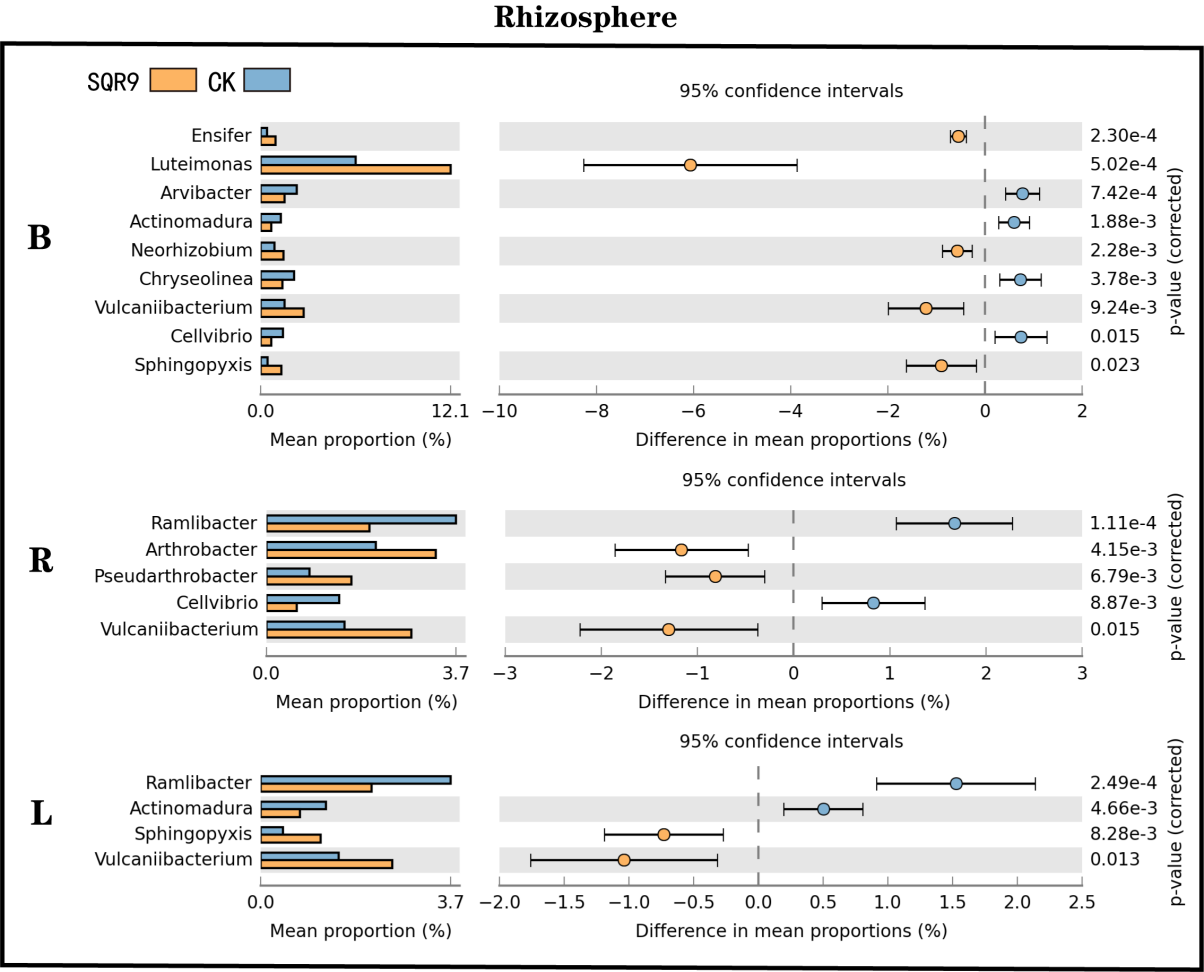
Difference analysis of the bacterial communities from the rhizosphere at the genus level. operational taxonomic units (OTUs) with abundances greater than 0.1% were selected to perform the analysis. The results show differential bacterial taxa with significance between CK and other treatments (*p* ≤ 0.05, fold change >1.5).

We further investigated the bacterial composition of the rhizosphere and phyllosphere at the phylum level and genus level. Compared with the CK, the application of SQR9 increased Actinobacteria and Firmicutes and decreased Proteobacteria and Bacteroidetes in the phyllosphere. However, the composition of the rhizosphere at the phylum level was relatively stable (Figure S4a,b). Moreover, based on the difference analysis of bacterial community composition at the genus level, we found that all the treatments with applied SQR9 significantly increased *Vulcaniibacterium* and *Rhizobium* compared with those in the CK rhizosphere (Figure 2, Figures S9, S10 and S11), and the relative abundance of *Cutibacterium* was increased in most of the SQR9 application treatments compared with that in the CK phyllosphere (Figures S5, S6, S7 and S8). In addition, the relative abundances of *Luteimonas*, *Cellvibrio* and *Sphingopyxis* were increased in the rhizosphere in most of the SQR9 application treatments.

## DISCUSSION

The existence of PGPB in agricultural soil and its interaction with plants has received keen attention worldwide. However, the symbiotic nature of plant–microorganism interactions suggests that PGPB need not necessarily be inoculated into the soil to play a role (Trivedi et al., 2020). Some studies have shown that foliar spraying of PGPB also shows significant effects (Esitken et al., 2006), but these studies did not compare the corresponding behaviour of PGPB in the rhizosphere and leaves. Therefore, we focused on this omission in our study. The results showed that PGPB inoculation of both the soil and phyllosphere promoted plant growth, which indicates that the application methods of functional bacteria in agricultural production can be quite diverse.

The abundance of beneficial bacteria is normally related to the function of the community (Ling et al., 2022). The increased abundance of SQR9 in the compartments with PGPB application suggested that the inputs of beneficial bacteria are involved in enhanced growth‐promoting ability via direct effects on the plant (Wang et al., 2021). *B. velezensis* could also colonize plants endophytically and migrate from belowground to aboveground (Kang et al., 2018). Moreover, *Bacillus* spp. has been widely reported to have the ability to directly affect other microorganisms and induce plant immune responses, which can subsequently activate the recruitment of commensal microorganisms by plants through the secretion of diverse metabolites (Saxena et al., 2020). Therefore, the application of SQR9, regardless of where it was applied, changed the bacterial composition of both the rhizosphere and phyllosphere in this study. In addition, the bacterial community from the rhizosphere showed a greater contribution to plant growth than that from the phyllosphere, and more potentially functional taxa were also observed in the rhizosphere than in the phyllosphere. This phenomenon may be related to the high diversity of rhizosphere microorganisms, which leads to potentially high plant growth‐promoting ability (Ling et al., 2022). The foliar spray of PGPB stimulated plants to recruit potential functional taxa similar to those in *Bacillus* soil inoculation such as *Rhizobium, Luteimonas* and *Sphingopyxis*, and these taxonomy have been widely reported to promote plant growth through increasing the acquisition of nitrogen and/or secreting IAA (Dias et al., 2009; Ulrich et al., 2022; Yang et al., 2022). Thus, we speculated that PGPB inoculation, both in the soil and phyllosphere, can directly colonize and indirectly stimulate beneficial microorganisms in the rhizosphere to promote plant growth.

**FIGURE 3:**
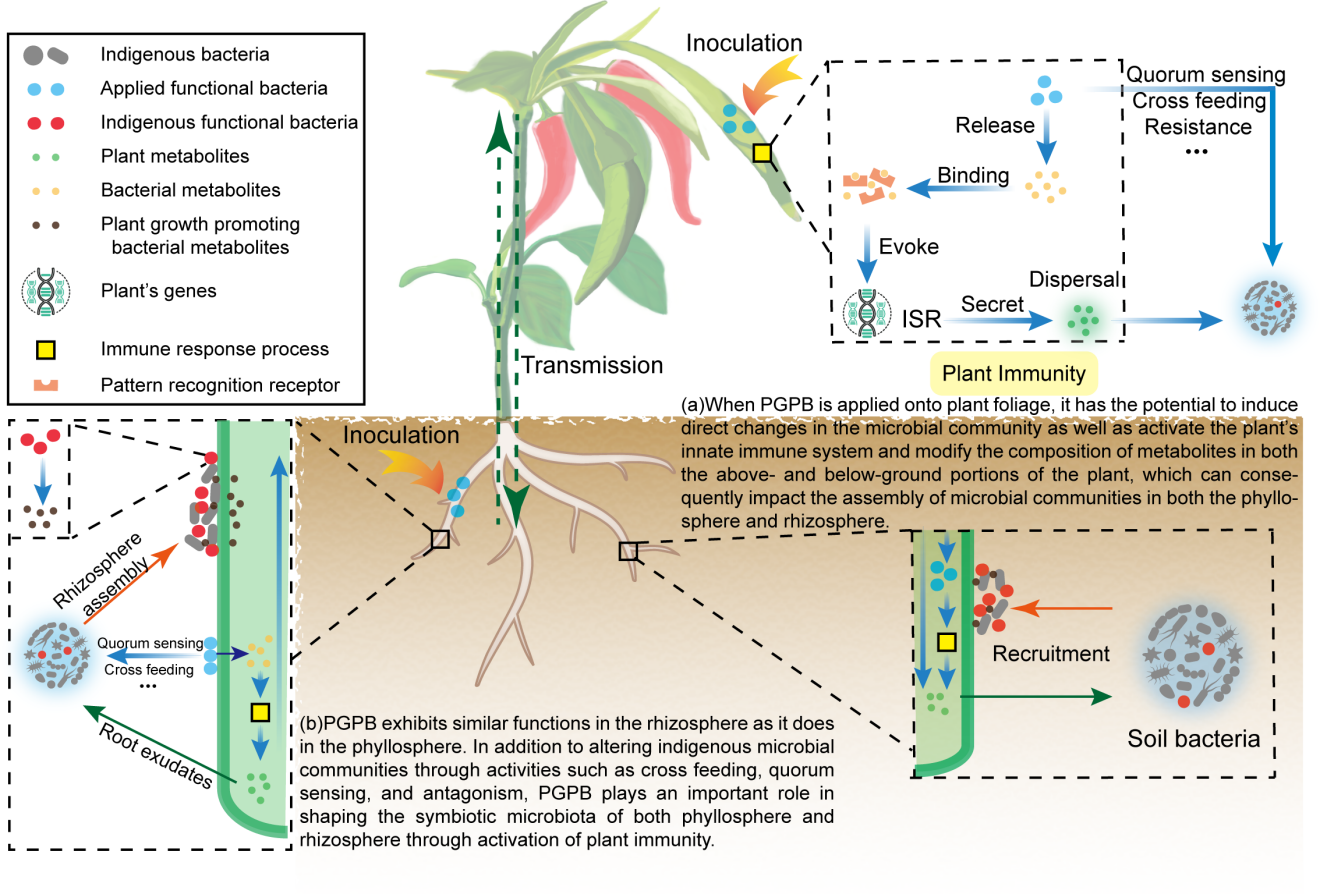
Conceptual model of the plant growth‐promoting process under plant growth‐promoting bacteria (PGPB) phyllosphere inoculation.

Based on the results presented above and existing reports, we propose that foliar spraying of PGPB promotes plant growth via direct and indirect mechanisms in the following manner, as summarized in Figure 3. When PGPB are inoculated into plant foliage, they are rapidly targeted by the plant innate immune system, priming induced systemic resistance (ISR) and triggering JA‐, SA‐ and ET‐related signalling pathways and consequently influencing microbial diversity and assembly in the phyllosphere (Jones & Dangl, 2006; Shakir et al., 2021). Together with bacteria, the immune molecules, and related metabolites produced in response to inoculation then diffuse vertically through the vessels from the aboveground parts to the roots (Compant et al., 2008; Shakir et al., 2021). These changes in the roots affect the composition of the root exudates, including carbohydrates and amino acids (Shakir et al., 2021; Trivedi et al., 2020). Plant‐associated microorganisms are attracted to the signals and therefore accumulate in the rhizosphere (Scharf et al., 2016). Similarly, PGPB colonization in the rhizosphere could also affect bacterial assembly in both the rhizosphere and the phyllosphere to promote plant growth. Therefore, this research points to the complex interactions between plants and their associated microbiomes and could refine the selection and use of PGPBs in different agricultural settings. On the one hand, the mention of vertical diffusion could incite research into the connectivity between aboveground and belowground microbial communities. Understanding this relationship could be key to holistic approaches to plant growth, health and soil management. On the other hand, the changes in root exudate composition influenced by foliar‐sprayed PGPB suggest that research could be directed toward understanding how these changes influence the soil microbiota and plant health, potentially leading to the development of tailor‐made PGPB treatments based on root exudate profiles. Integrating foliar spraying of PGPB into agricultural practices could improve crop yields through the potential mechanisms described above. However, in future research, there is still a need for more work on studying specific microorganisms that induce plant immune responses and root exudates and exploring the microbial‐induced changes in both aboveground and underground transmission. Overall, continued research in these areas will contribute to the development of sustainable agricultural practices and the promotion of plant growth in the future.

In summary, the results of this study confirmed that foliar spraying of PGPB could promote plant growth similar to traditional bulk and rhizosphere applications. PGPB, regardless of where it was inoculated, led to a shift in the bacterial communities in both the rhizosphere and phyllosphere. Specifically, similar beneficial microorganisms, such as *Rhizobium*, *Luteimonas* and *Sphingopyxis*, were induced by PGPB, potentially through aboveground and belowground vertical conversation, in all plant compartments to promote plant growth. Notably, the PGPB‐mediated rhizosphere–phyllosphere interaction remains a subject for future study to elucidate its role in plant growth promotion and permit the design of better agricultural management.

## AUTHOR CONTRIBUTIONS

**Zhao Yu:** Investigation (equal); methodology (equal); visualization (equal); writing – original draft (lead). **Dongsheng Wang:** Data curation (equal); investigation (equal); methodology (equal); visualization (equal). **Bo Zhang:** Investigation (supporting); methodology (supporting). **Hancheng Mao:** Investigation (supporting); methodology (supporting). **Zhe Wang:** Investigation (supporting); methodology (supporting). **Zhiguang Yan:** Investigation (supporting); methodology (supporting). **Chengyuan Tao:** Conceptualization (supporting); methodology (supporting); visualization (equal). **Xuhui Deng:** Conceptualization (equal); investigation (supporting); supervision (equal); visualization (supporting); writing – review and editing (equal). **Qirong Shen:** Conceptualization (supporting); supervision (equal). **Rong Li:** Conceptualization (equal); supervision (equal); writing – review and editing (supporting).

## CONFLICT OF INTEREST STATEMENT

None declared.

## Supporting information


**Data S1.** Supporting Information.

## Data Availability

The raw sequence data for the 16S amplicon sequencing of all samples are available in the NCBI Sequence Read Archive database with the accession number SRP431257.
