# Supplementary material for: Bacillus velezensis SQR9 promotes plant growth through colonization and rhizosphere–phyllosphere bacteria interaction
Source: Environ Microbiol Rep. 2024 Apr 4;16(2):e13250. doi: 10.1111/1758-2229.13250 (PMC10994692; doi:10.1111/1758-2229.13250)
Supplement: Supplementary file 1 — Data S1. Supporting Information. [file EMI4-16-e13250-s001.docx]

**Supplementary figures:**


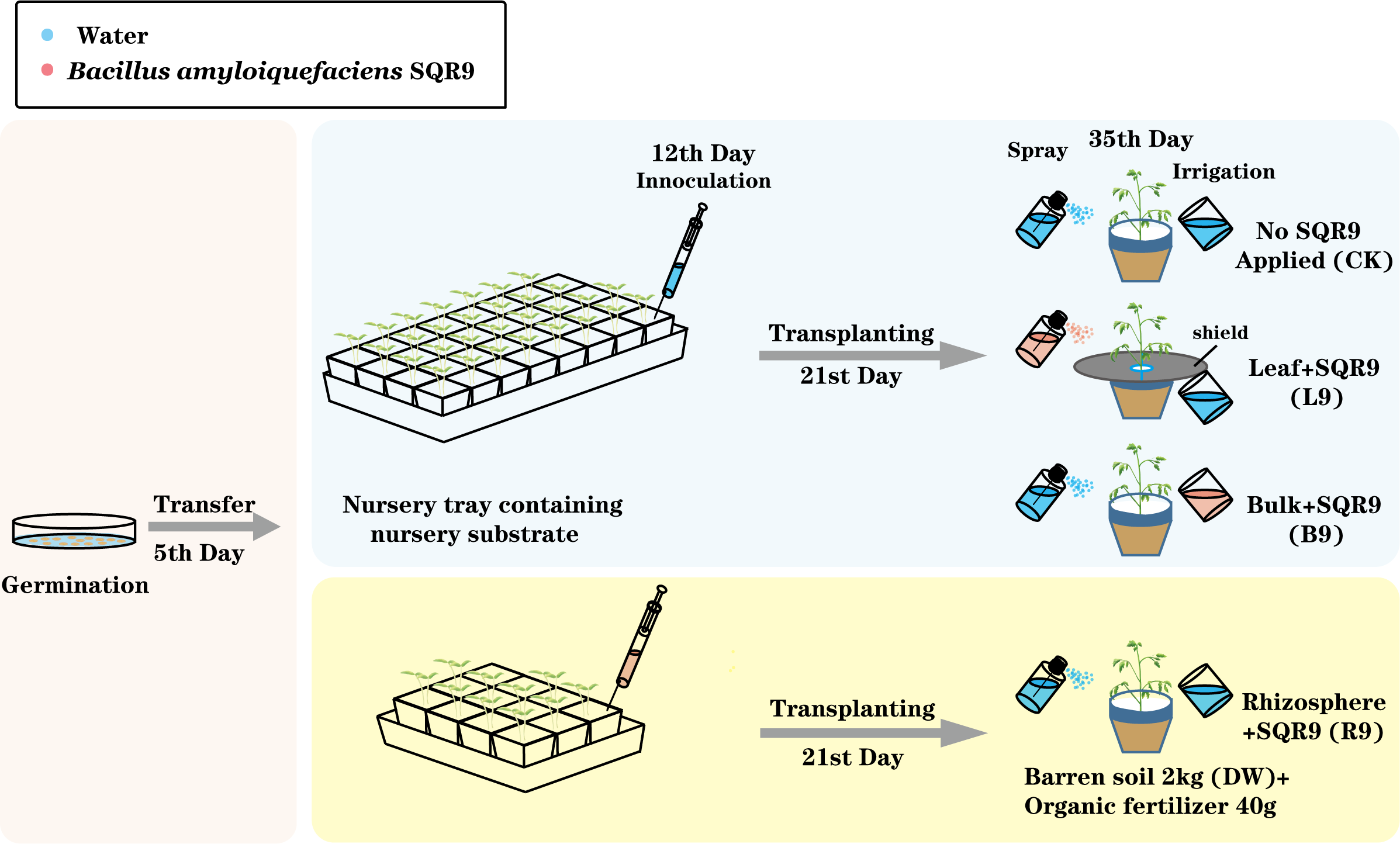


**Fig. S1** Experimental flow for this study.


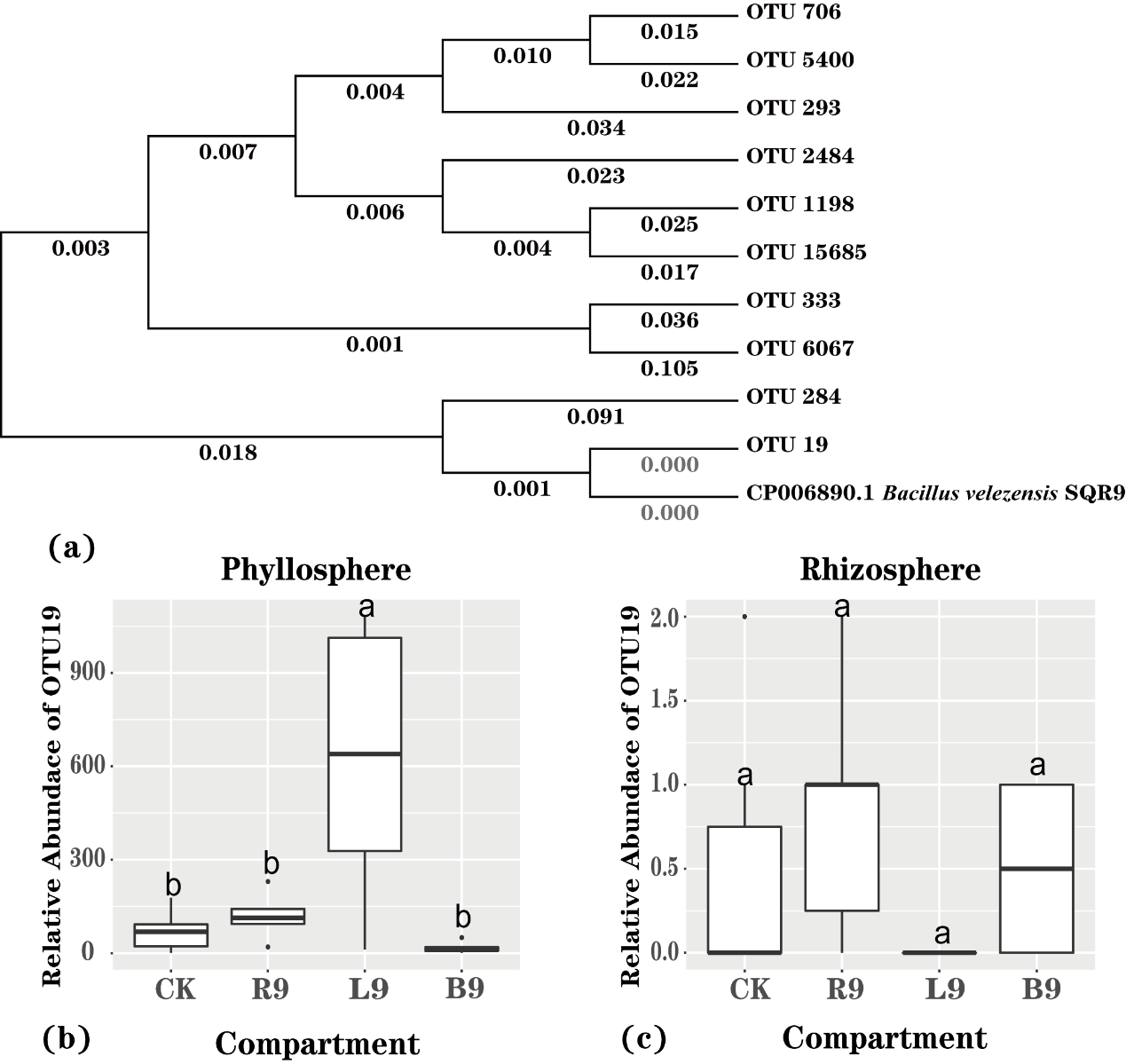


**Fig. S2** The influence of SQR9 application on the bacterial community. (a) Neighbour-joining phylogenetic tree based on 16S rRNA gene sequences showing the relationship of strain OTU19 to closely related type strains. (b) Relative abundance of OTU19 in the phyllosphere (mean±SE). (c) Relative abundance of OTU19 in the rhizosphere (mean±SE). The values associated with branches in the phylogenetic tree represent the phylogenetic distance between two branches. CK, treatment without SQR9 application; R9, seedling growing medium with SQR9 application; L9, leaves were sprayed with SQR9; B9, SQR9 was added to bulk soil. R, rhizosphere; L, phyllosphere. Different letters indicate significant differences among the treatments as determined by Tukey’s test (p≤0.05).


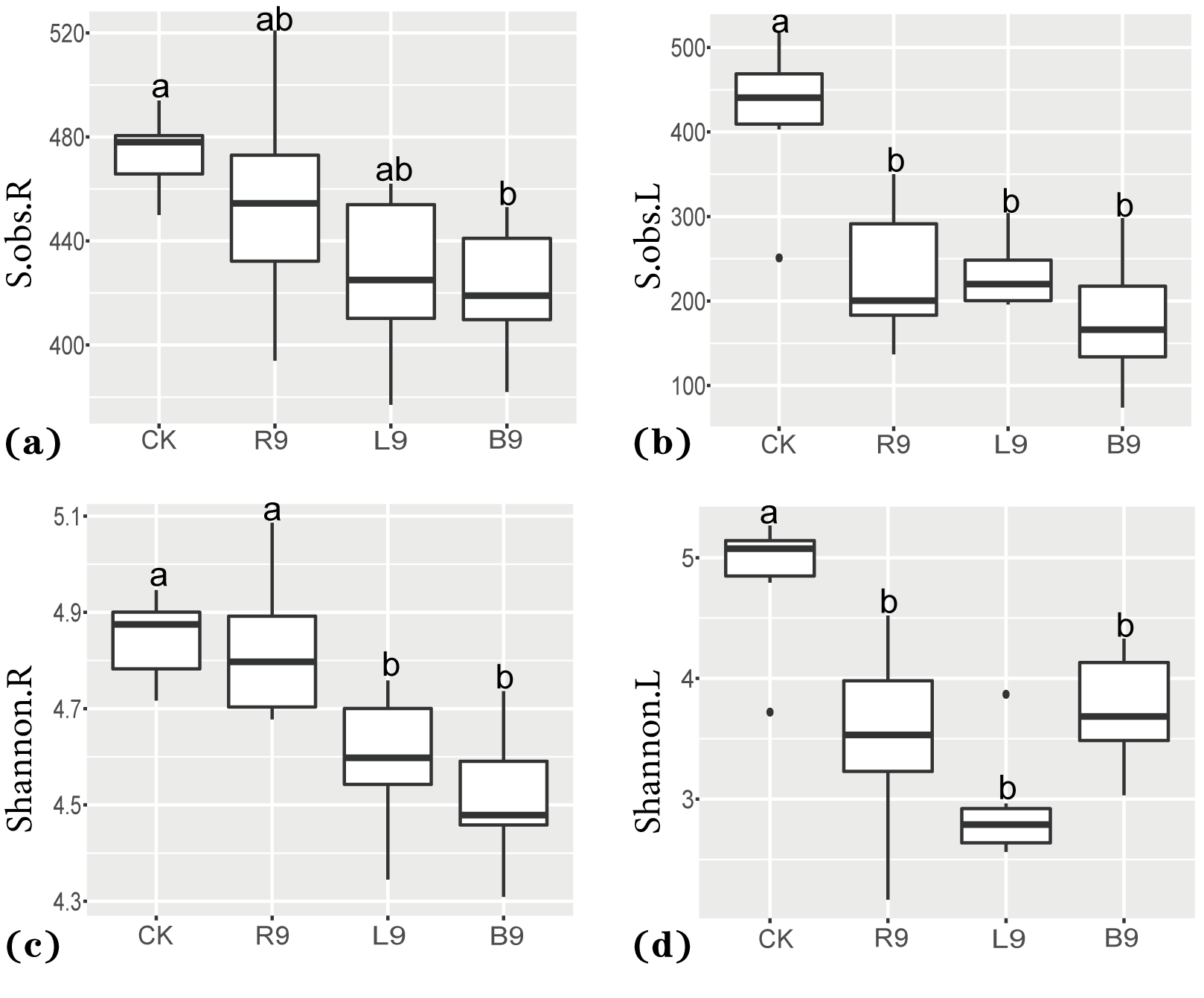


**Fig. S3** The influence of SQR9 application on the bacterial community. (a) Sobs richness of rhizosphere bacteria (mean±SE), (b) Sobs richness of phyllosphere bacteria (mean±SE), (c) Shannon diversity of rhizosphere bacteria (mean±SE), and (d) Shannon diversity of phyllosphere bacteria (mean±SE). CK, treatment without SQR9 application; R9, seedling growing medium with SQR9 application; L9, leaves were sprayed with SQR9; and B9, SQR9 was added to bulk soil. R, rhizosphere; L, phyllosphere. Different letters indicate significant differences among the treatments as determined by Tukey’s test (p≤0.05).


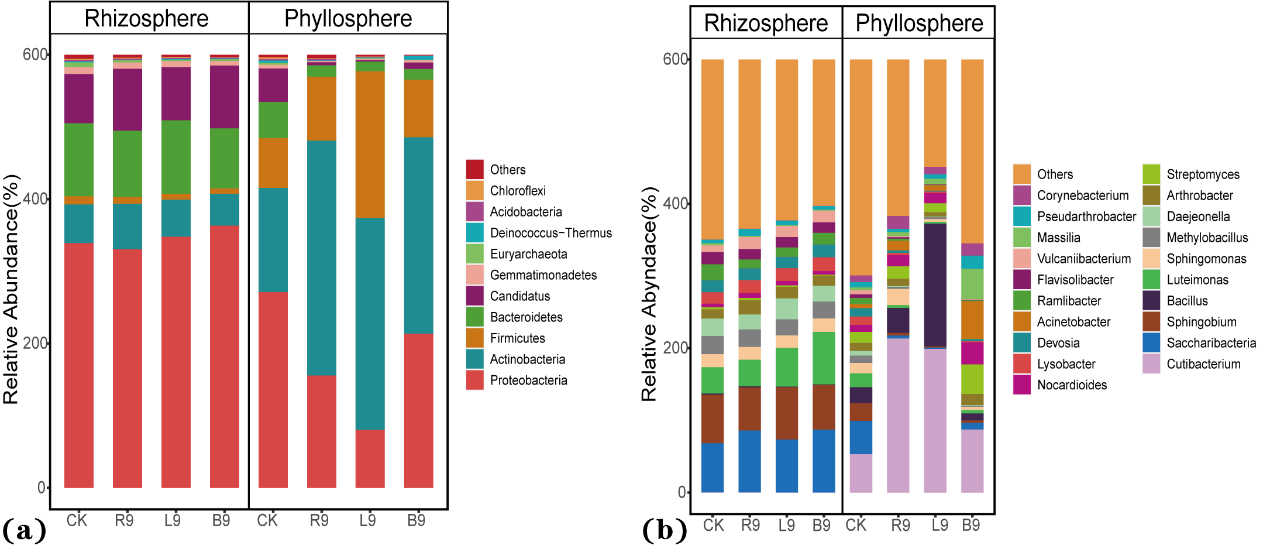


**Fig. S4** The influence of SQR9 application on the bacterial community. (a) Taxonomic features of the top 10 abundant phyla in rhizosphere and phyllosphere bacterial communities under different treatments. “Others” denotes rare phyla. (b) Taxonomic features of the top 20 abundant genera in rhizosphere and phyllosphere bacterial communities under different treatments. “Others” denotes rare genera. CK, treatment without SQR9 application; R9, seedling growing medium with SQR9 application; L9, leaves were sprayed with SQR9; and B9, SQR9 was added to bulk soil. R, rhizosphere; L, phyllosphere.


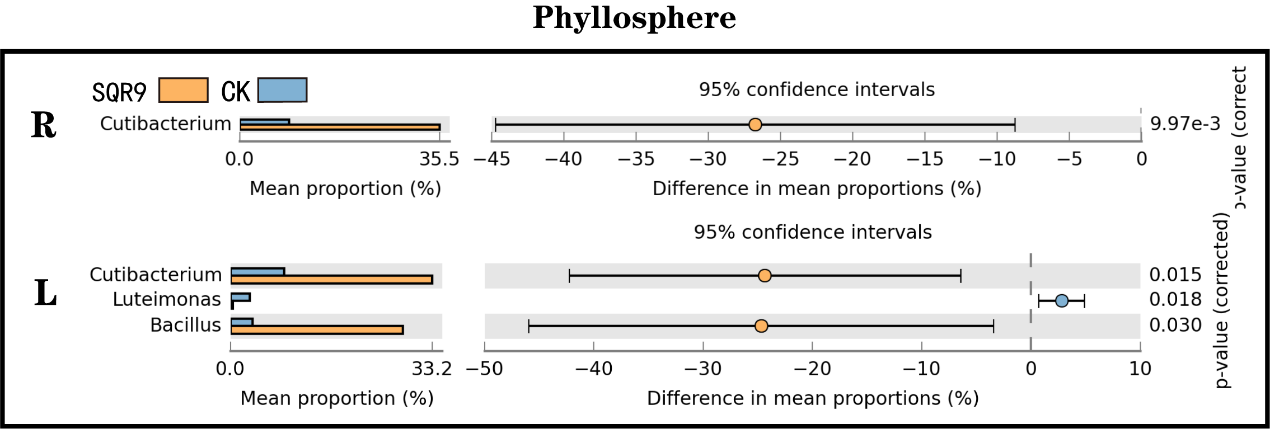


**Fig. S5** Difference analysis of the bacterial communities from the phyllosphere at the genus level. OTUs with abundances greater than 0.1% were selected to perform the analysis. The results show differential bacterial taxa with significance between CK and other treatments (p ≤ 0.05, fold change > 1.5).


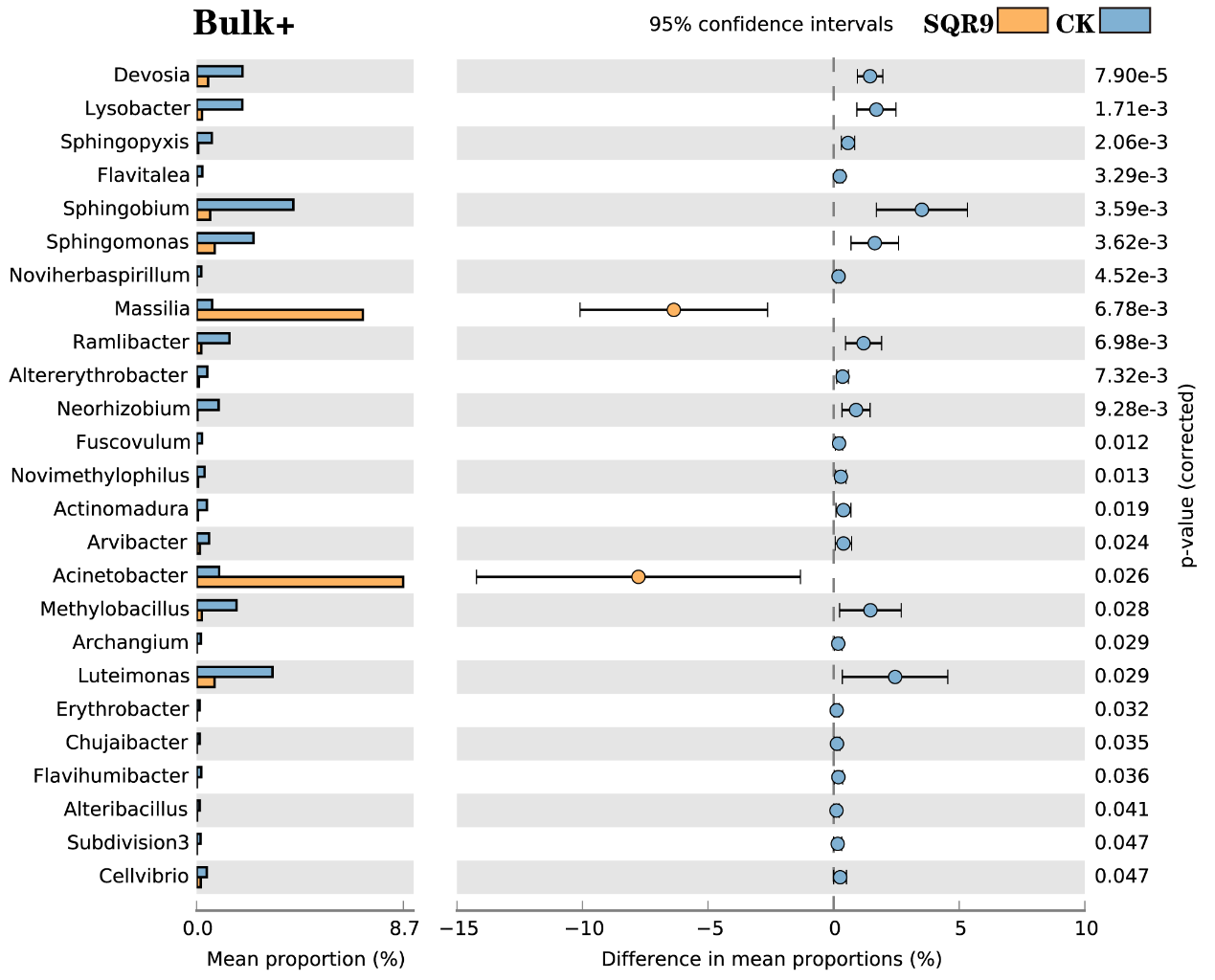


**Fig. S6** Difference analysis of the bacterial communities between the bulk soil application and control treatments from the phyllosphere at the genus level. OTUs with abundances greater than 0.1% were selected to perform the analysis. The results show differential bacterial taxa with significance between CK and other treatments (p ≤ 0.05).


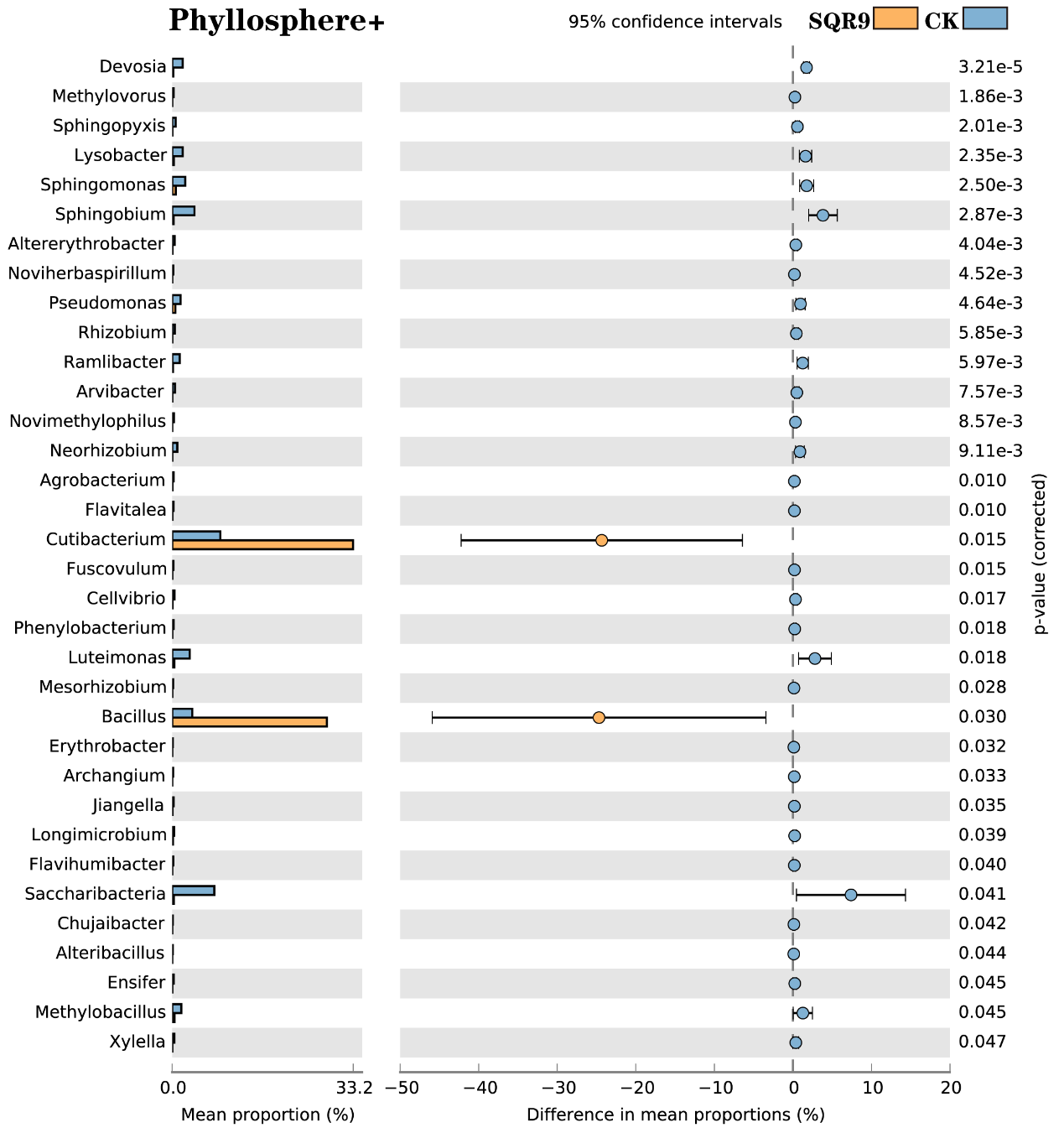


**Fig. S7** Difference analysis of the bacterial communities between the phyllosphere application and control treatments from the phyllosphere at the genus level. OTUs with abundances greater than 0.1% were selected to perform the analysis. The results show differential bacterial taxa with significance between CK and other treatments (p ≤ 0.05).


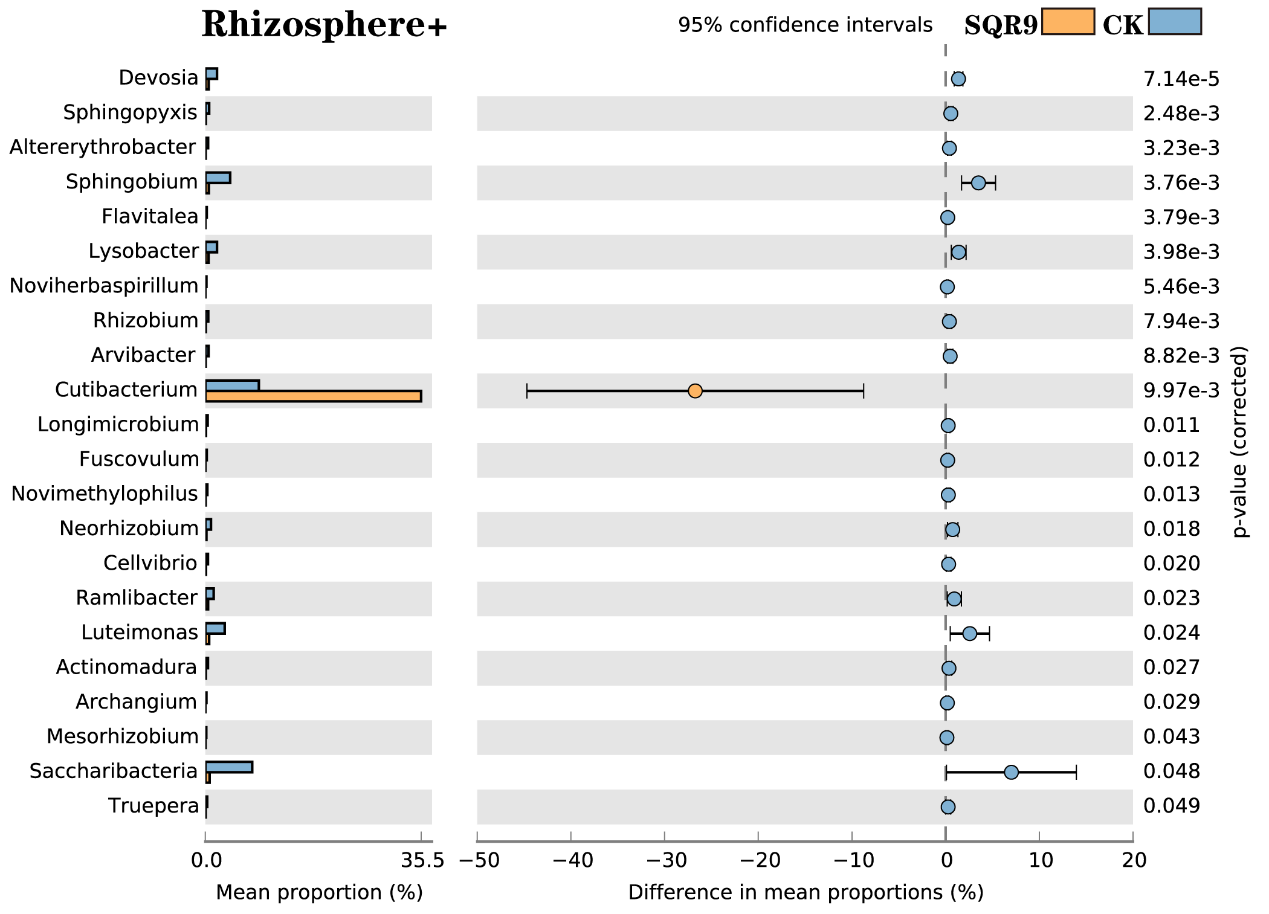


**Fig. S8** Difference analysis of the bacterial communities between the rhizosphere application and control treatments from the phyllosphere at the genus level. OTUs with abundances greater than 0.1% were selected to perform the analysis. The results show differential bacterial taxa with significance between CK and other treatments (p ≤ 0.05).


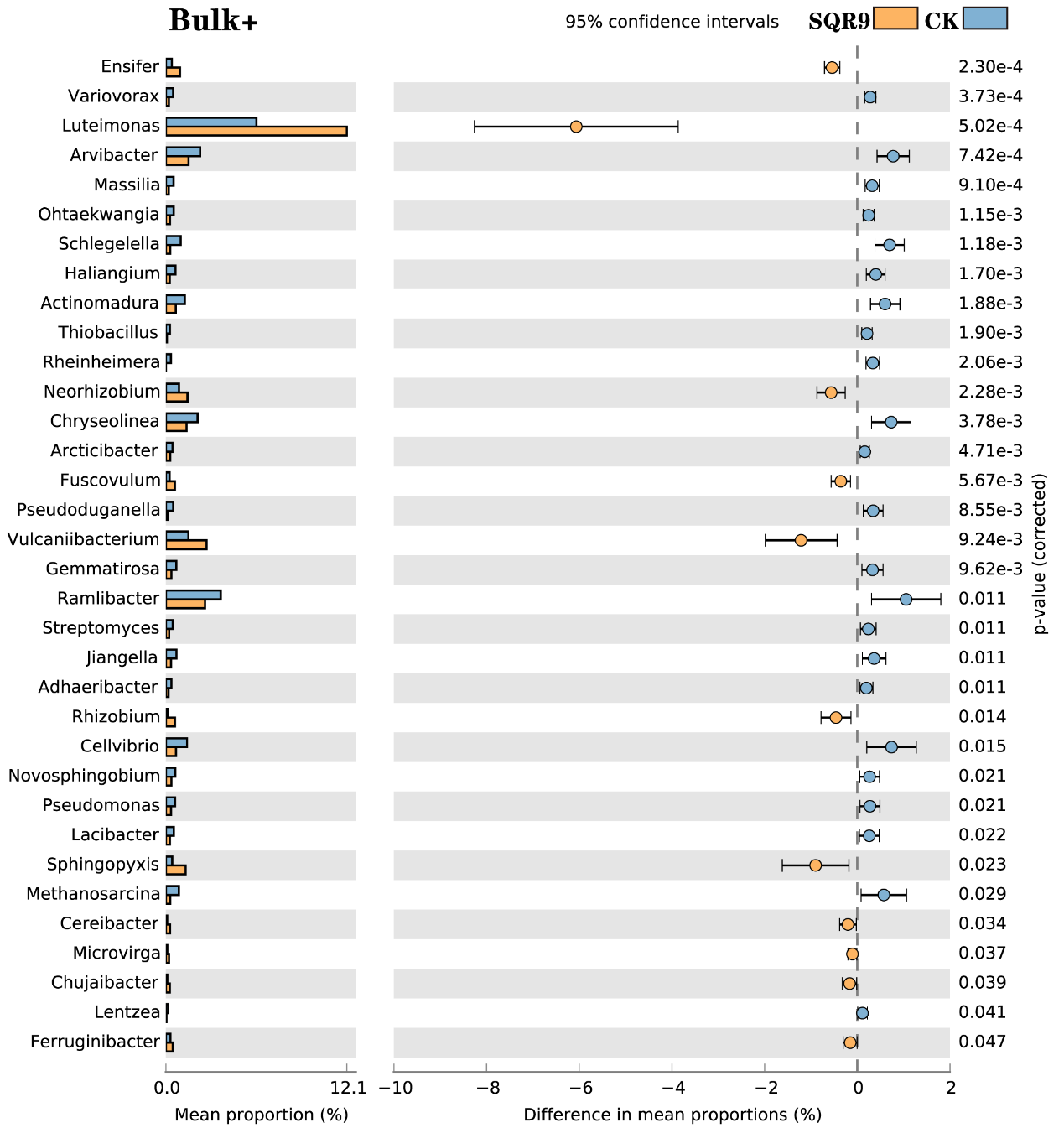


**Fig. S9** Difference analysis of the bacterial communities between the bulk soil application and control treatments from the rhizosphere at the genus level. OTUs with abundances greater than 0.1% were selected to perform the analysis. The results show differential bacterial taxa with significance between CK and other treatments (p ≤ 0.05).


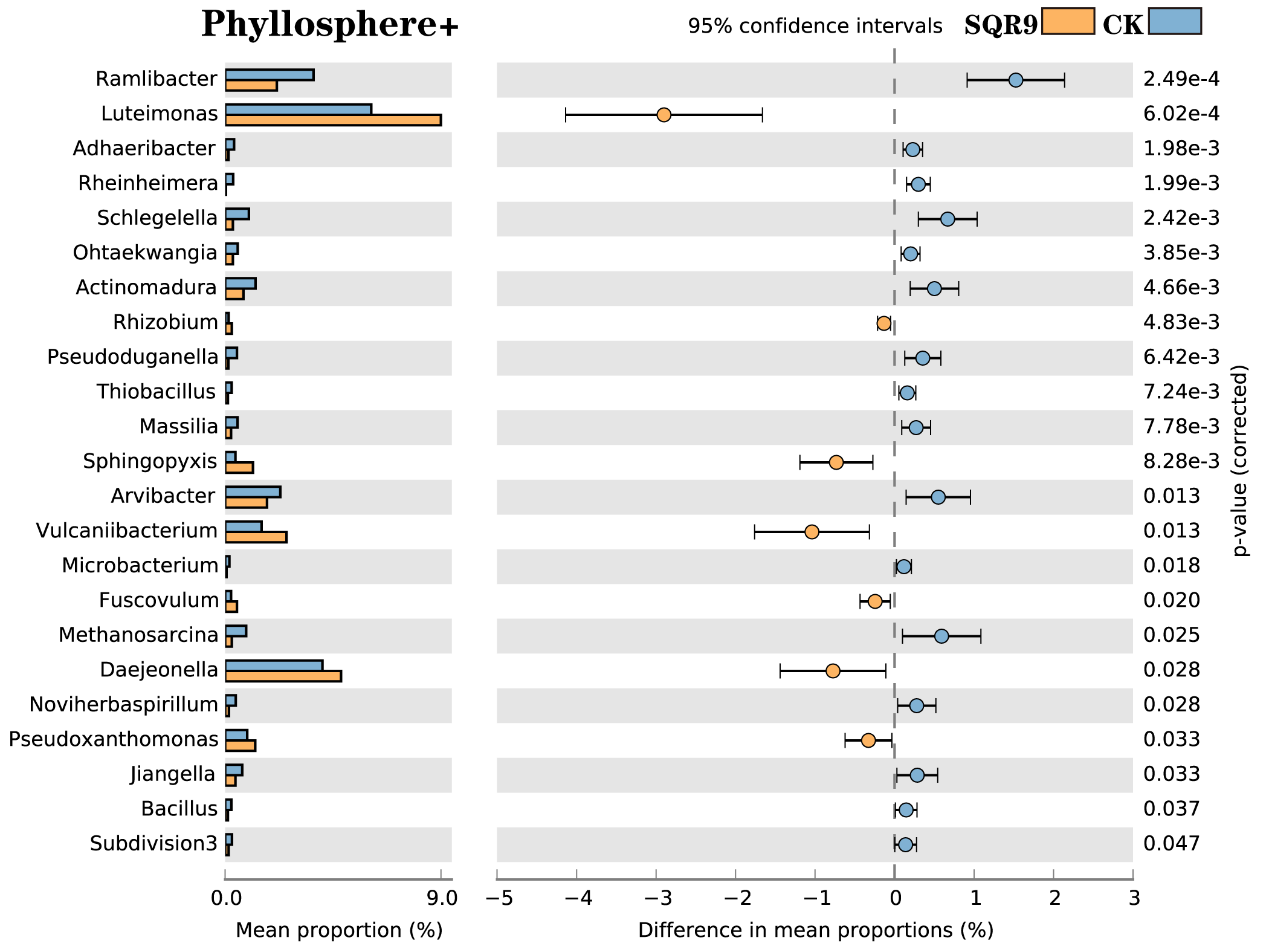


**Fig. S10** Difference analysis of the bacterial communities between the phyllosphere application and control treatments from the rhizosphere at the genus level. OTUs with abundances greater than 0.1% were selected to perform the analysis. The results show differential bacterial taxa with significance between CK and other treatments (p ≤ 0.05).


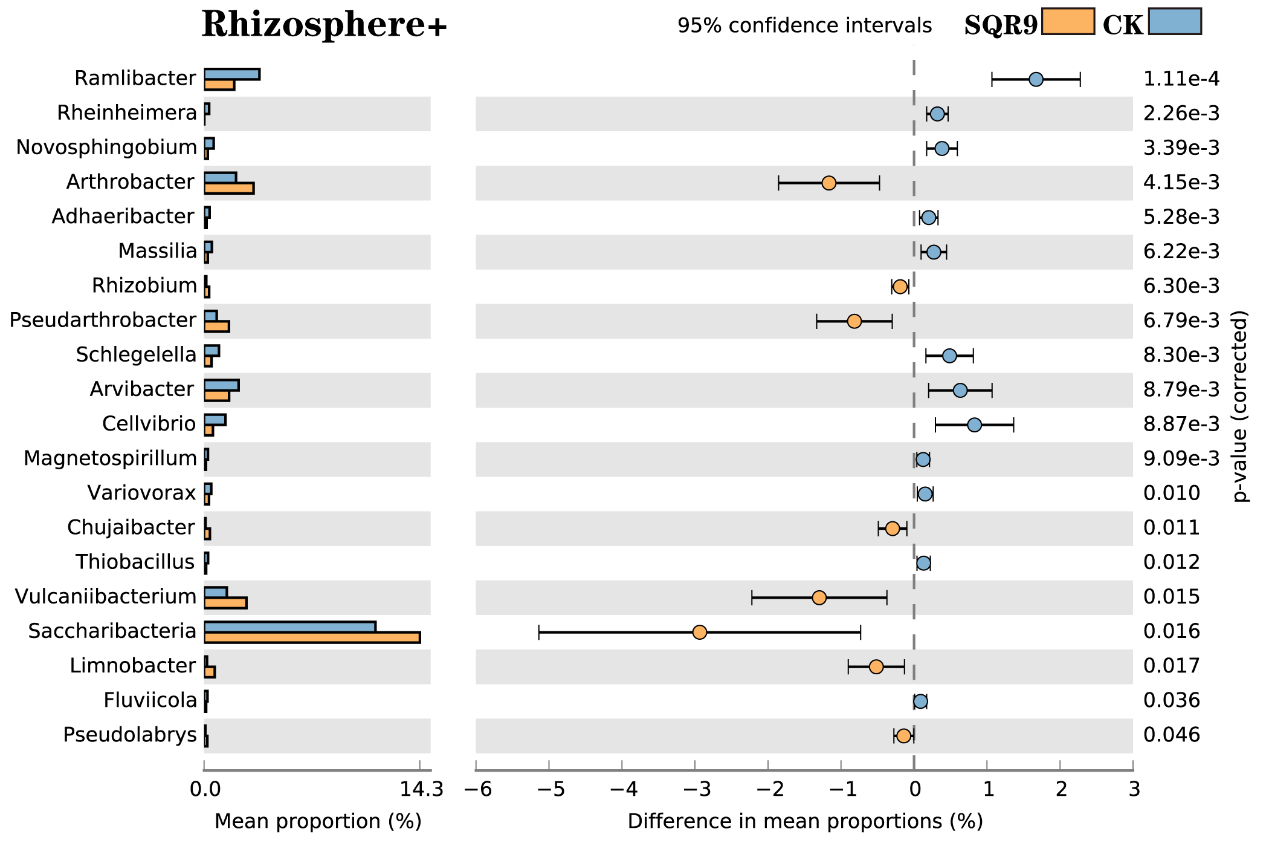


**Fig. S11** Difference analysis of the bacterial communities between the rhizosphere application and control treatments from the rhizosphere at the genus level. OTUs with abundances greater than 0.1% were selected to perform the analysis. The results show differential bacterial taxa with significance between CK and other treatments (p ≤ 0.05).
